# Antimicrobial resistance characteristics and fitness of Gram-negative fecal bacteria from volunteers treated with minocycline or amoxicillin

**DOI:** 10.3389/fmicb.2014.00722

**Published:** 2014-12-17

**Authors:** Miranda Kirchner, Muriel Mafura, Theresa Hunt, Manal Abu-Oun, Javier Nunez-Garcia, Yanmin Hu, Jan Weile, Anthony Coates, Roderick Card, Muna F. Anjum

**Affiliations:** ^1^Department of Bacteriology, Animal and Plant Health AgencyAddlestone, UK; ^2^Specialist Scientific Services Department, Animal and Plant Health AgencyAddlestone, UK; ^3^Department of Medical Microbiology, Institute of Infection and Immunity, St. George's University of LondonLondon, UK; ^4^Institute for Laboratory and Transfusion Medicine at the Heart and Diabetes Centre NRW, University Hospital of the Ruhr UniversityBochum, Germany

**Keywords:** antibiotic trial, resistance genes, facultative anaerobic Gram-negative bacteria, persistent, transient

## Abstract

A yearlong study was performed to examine the effect of antibiotic administration on the bacterial gut flora. Gram-negative facultative anaerobic bacteria were recovered from the feces of healthy adult volunteers administered amoxicillin, minocycline or placebo, and changes determined in antimicrobial resistance (AMR) gene carriage. Seventy percent of the 1039 facultative anaerobic isolates recovered were identified by MALDI-TOF as *Escherichia coli*. A microarray used to determine virulence and resistance gene carriage demonstrated that AMR genes were widespread in all administration groups, with the most common resistance genes being *bla*_TEM_, *dfr, strB, tet*(A), and *tet*(B). Following amoxicillin administration, an increase in the proportion of amoxicillin resistant *E. coli* and a three-fold increase in the levels of *bla*_TEM_ gene carriage was observed, an effect not observed in the other two treatment groups. Detection of virulence genes, including *stx1A*, indicated not all *E. coli* were innocuous commensals. Approximately 150 *E. coli* collected from 6 participants were selected for pulse field gel electrophoresis (PFGE), and a subset used for characterisation of plasmids and Phenotypic Microarrays (PM). PFGE indicated some *E. coli* clones had persisted in volunteers for up to 1 year, while others were transient. Although there were no unique characteristics associated with plasmids from persistent or transient isolates, PM assays showed transient isolates had greater adaptability to a range of antiseptic biocides and tetracycline; characteristics which were lost in some, but not all persistent isolates. This study indicates healthy individuals carry bacteria harboring resistance to a variety of antibiotics and biocides in their intestinal tract. Antibiotic administration can have a temporary effect of selecting bacteria, showing co-resistance to multiple antibiotics, some of which can persist within the gut for up to 1 year.

## Introduction

The use of antibiotics has played a crucial role in the acquisition and proliferation of antimicrobial resistance. A study of historical plasmids pre and post antibiotic use has demonstrated that prior to antibiotic utilization many plasmids did not carry these resistance genes (Hughes and Datta, 1983). However, the pressure exerted by the use of antibiotics has caused bacteria to adapt and acquire these elements. Frequently these elements are acquired from organisms in the environment, which possess them as a means of protection from natural antibiotics produced by bacteria inhabiting these niches (Martinez, 2008).

Antimicrobial resistance can be acquired by acquisition of novel genetic material on mobile elements such as plasmids, transposons, insertion sequences and, integrons. All play a role in the spread of resistances seen today. Plasmids have been studied widely and can carry multiple resistance genes resulting in their co-selection by several agents. For example, the plasmids encoding *bla*_CTX−M_ genes frequently encode additional resistance genes generating multi-drug resistance plasmids, which have become widespread and problematic. In addition, many plasmids also carry toxin-antitoxin systems which enable plasmid maintenance in bacterial cells, in the absence of an antibiotic (Carattoli, 2013).

The commensal bacterial population of the human intestine is a complex ecosystem. It is thought that the commensal bacterial population can act as a reservoir of antimicrobial resistance genes that can be disseminated on mobile elements to pathogenic bacteria and, between bacterial species (Marshall et al., 2009). In healthy individuals the gut resistome has been shown to carry a large pool of antibiotic resistance genes (Sommer et al., 2009), which could act as a reservoir and spread to bacterial pathogens that may also be present in the gut. Colonization with resistant bacteria can occur at an early age during the first weeks after birth, in the absence of antibiotic use, due to acquisition from the mother or environment (Zhang et al., 2011). Evidence indicates that the acquisition of resistance genes or the resistome in an individual can occur without the need for antibiotic use due to the abundance of mobile elements harboring these genes (Zhang et al., 2011), and that resistance genes are often maintained without the need for selective antibiotic pressure (Salyers et al., 2004).

In this study we describe the results of a human trial performed to assess the effect of antimicrobial use on facultative anaerobic Gram-negative bacteria in the gut, bacteria which commonly harbor transferable resistance elements. The isolates were collected as part of the Anitresdev FP7 project (http://www.ucl.ac.uk/antiresdev). The results from the obligate anaerobic Gram-negative isolates collected from this study have previously been reported (Kirchner et al., 2013). A single course of either an antibiotic (ampicillin or minocycline), or a placebo, was administered to between 10 and 15 healthy adult participants per group and they were then sampled periodically over 1 year. Amoxicillin and Minocycline were chosen as they are frequently prescribed antimicrobial agents which are of different antibiotic classes. They also act in different ways with amoxicillin having bactericidal activity, while minocycline is bacteriostatic. The aim was to determine if the use of an antibiotic altered the antibiotic resistance gene carriage in Gram-negative facultative anaerobes present in the intestinal flora. Unlike many resistome and microbiome study previously published (Moore et al., 2013; Card et al., 2014), we assessed resistance gene carriage in individual bacteria rather than in the whole gut bacterial population. This allowed us to determine the types of bacteria present in the gut that are most likely to carry resistance genes and is detectable by culture methods used in this study; to identify the most common resistance determinants they carry, and whether the presence of one resistance gene may be linked with another. The study also assessed whether there are differences between *Escherichia coli* isolated from these participants at multiple visits (considered persistent), and those detected at a single visit (considered transient), by using both molecular profiling and response to stress modulators.

## Methods

### Challenge study and isolate selection

A total of 44 healthy subjects (18–40 years old) were recruited for this study. The study was conducted in healthy volunteers from the general public in South West London, UK, between 2010 and 2011. It was designed as an open labeled parallel group clinical trial. The study was approved by the South West London Research Ethics Committee (Reference 10/h0806/12) of St George's University of London and the Medicines and Healthcare products Regulatory Agency (2009-017647-34), UK. The clinical trial was registered in a public registry (study ID number C09040). Written consent was obtained from all the volunteers that agreed to participate in this study.

There were 15 subjects allocated to each of the cohorts administered amoxicillin and minocycline, and 14 subjects in the placebo cohort. The dosage used was that recommended by the British National Formulary for amoxicillin 250 mg (Amoxil capsules, GlaxoSmithKline, UK), three times daily for 7 days; and minocycline 100 mg (Aknemin capsules, Almirall), twice daily for 5 days. Placebo was given to the control group. Ten subjects withdrew due to adverse effects and personal reasons; which included 3 from the amoxicillin group, 5 from minocycline group and 2 from the placebo group. Fecal samples were collected from each volunteer at day 0, before antibiotic administration; at day 11, immediately after treatment completion; then at 30, 60, 120, 356 days following treatment completion (6 sampling points in total). Fecal samples obtained from participants from all groups were serially diluted on to blood agar containing 0.5 mg/L minocycline, 4 mg/L minocycline, and 8 mg/L amoxicillin to isolate resistant bacteria, as previously described (Nord et al., 2006). The number of isolates selected from each participant at each sampling point was dependant on the number and type of colonies observed on the 3 plates. After incubation, up to 5 different colony types were picked from each plate, sub-cultured to purity and stored at −70°C for further analysis. Therefore, for each participant up to 5 colonies or isolates were selected from up to 3 antibiotic containing plate i.e., up to 15 isolates per sampling point. This resulted in a total of 1039 facultative anaerobic Gram-negative isolates being selected across all 6 sampling points from 34 volunteers.

### Bacterial isolates and identification

Purified isolates received at the Animal and Plant Health Agency (APHA) were cultured on sheep blood agar plates overnight at 37°C. The bacterial identity was determined using Matrix-assisted laser desorption/ionization time- of-flight (MALDI-TOF) (Carbonnelle et al., 2011).

### Antimicrobial resistance gene array analysis

A DNA microarray containing probes to 75 resistance genes encompassing 19 different antimicrobial classes (Batchelor et al., 2008; Anjum et al., 2011; Card et al., 2013) was used to test all isolates. For DNA extraction, each strain was grown overnight on Luria Bertani (LB) agar plates. DNA was extracted by re-suspending a loopful of fresh growth in 400 μl of lysis buffer (containing 0.24 mg/l proteinase K, 0.05% Tween 20, 0.1 M Trizma-HCl), mixed and incubated in a Thermomixer for 2–4 h at 60°C with agitation at 550 rpm, followed by 95°C for 15 min. Cell debris was removed following centrifugation and supernatant used in subsequent steps. The method and primers used for labeling of DNA present in the supernatant are as described previously (Batchelor et al., 2008; Anjum et al., 2011; Card et al., 2013). Labeled DNA was hybridized to the array using the buffers provided in the HybPlus Kit (Alere Technologies, Jena, Germany). For all hybridization and washing steps the arrays were agitated at 550 rpm in a Thermomixer. Hybridization was performed at 55°C for 1 h and washing at 30°C. Horse-radish peroxidase conjugated Streptavidin was added for 15 min at 30°C and bound DNA was detected using Seramun Green (Seramun Diagnostica, GmbH, Germany). Signals were read using an ArrayMate scanner and IconoClust software (Alere Technologies). Mean signal intensities of two replicate spots per probe were used for analysis and values ≥0.5 were considered positive.

To determine the number of isolates that were multi-drug resistant (MDR) based on gene carriage, resistance genes were grouped according to their mode of activity (as classified previously, Card et al., 2013) and the number of positive groups in each isolate determined. Integrase genes were not included as they do not encode for resistance activity. Isolates that were positive for resistance genes in two or more antibiotic classes were defined as MDR.

### Pulsed field gel electrophoresis

Pulsed-field gel electrophoresis (PFGE) using *Xba*I restriction endonuclease was performed for selected *E. coli* isolates according to the PulseNet protocol for *E. coli* O157 (Centers for Disease Control and Prevention, Atlanta, USA; http://www.cdc.gov/pulsenet/pathogens/index.html). Clustering analysis was performed using BioNumerics (Applied Math). Dendrograms were generated using Gel Compare and banding patterns analyzed using DICE co-efficient and unweighted pair group method with arithmetic averages (UPGMA).

### Plasmid analysis and transferability studies

Plasmid DNA was extracted using a method described previously (Kado and Liu, 1981; Liebana et al., 2004). Plasmids were analyzed using BioNumerics to estimate their size; *E. coli* strain 39R861 containing plasmids of known sizes (148, 63.4, 36, and 6.9 kb) and a supercoiled DNA Ladder (Sigma-Aldrich, UK), were used for comparison.

To identify the addiction systems, which are a toxin-antitoxin system carried by plasmids for maintenance in bacteria, a PCR method described previously was used (Mnif et al., 2010). Seven PCRs were used to detect, *pemk, hok-sok, ccdAB, relBE, vagCD, pndAC*, and *srnBC*. A PCR based method was also used to determine the incompatibility groups of the plasmids carried by these isolates. A multiplex PCR kit from Diatheva (Fano, Italy), which is based on a previous publication (Carattoli et al., 2005), was used. The kit was used according to the manufacturer's instructions.

An in-broth conjugation method was used to isolate conjugative plasmids carried by these *E. coli*. *Salmonella* Typhimurium strain 26R775 was used as the recipient for these studies. Each strain was grown overnight at 37°C in static conditions in 3 ml LB broth. Conjugal-transfer was assessed after 5 and 24 h incubation by plating on Rambach containing 100 mg/l rifampicin, and either 16 mg/l ampicillin, 15 mg/l tetracycline, or 8 mg/l streptomycin, depending on the resistance genes carried by the parent strain. Following overnight incubation at 37°C, plates were enumerated and, conjugation rates determined where transfer occurred.

### Multi-locus sequence typing (MLST)

A subset of 14 *E. coli* selected from PFGE, were analyzed by multi-locus sequencing typing (MLST). This was performed using the loci, *adk, fumC, gyrB, icd, mdh, purA*, and *recA*, according to published method (Wirth et al., 2006). Sequences were compared to the *E. coli* MLST database held at the University of Cork, Dublin (http://mlst.warwick.ac.uk/mlst/dbs/Ecoli/).

### Assessment of stress response mechanisms

For assessment of stress response, isolates were analyzed using the Biolog phenotype microarray (PM) system (Hayward, CA, USA), which enables a high throughput screen of bacterial response to metabolic effector compounds (Bochner et al., 2001). Each isolate was analyzed using 10 PM plates containing antimicrobials (PM11–14) and other metabolic effectors (PM15–20). All media, reagents, and PM panels were used according to manufacturer's instructions. Bacterial isolates to be tested were cultured for 16 h on LB agar plates at 37°C in aerobic conditions. Cells were re-suspended in 10 ml of inoculating fluid (IF-0a) and the optical density adjusted to 85% transmittance. For each plate 12 ml of media IF-10a was mixed with 120 μl of bacterial cell suspension; this was also supplemented with a final concentration of 0.02 M sodium succinate/0.002 mM ferric citrate. Tetrazolium dye A, supplied by Biolog Inc (Hayward, CA, USA), was used to measure the cellular respiration. All microplates were incubated in the Omnilog instrument at 37°C and monitored for color change at 15 min intervals for 120 h. Kinetic data was analyzed with OmniLog-PM software (Biolog). All experiments were performed in duplicate on different days and comparisons between strains were based on the average of the area under the curve (AUC) values at 24 h. A comparison of respiratory profiles was performed by hierarchical clustering and PCA analysis with AUC values using the program R (www.bioconductor.org/).

## Results

### Alterations in gene carriage in the bacterial population following antibiotic administration

#### Isolate characterization and resistance gene carriage

This work reports on Gram-negative fecal isolates that were collected over one year from 3 groups, at six different time points. The 3 groups included in the study were: amoxicillin administered; minocycline administered and placebo. All isolates grew under aerobic conditions, were Gram-negative bacteria, and belonged to 19 bacterial genera (Supplementary Table 1). Isolates were picked depending on their colony morphology after growth on selective media containing antibiotic; the total number of isolates collected at each sampling point from each participant varied from 0 to 15 and the total number isolated across all 3 groups and 6 sampling points, were 1039. There were 378 isolates from the amoxicillin administered group (133 from amoxicillin plates, 241 from minocycline plates, 4 were unknown), 248 isolates from the minocycline administered group (83 from amoxicillin plates, 162 from minocycline plates, 3 were unknown), and 413 isolates from the placebo (151 from amoxicillin plates, 256 from minocycline plates, 6 were unknown). Seventy percent of those tested were *E. coli* (*n* = 729), with the other major groups being *Klebsiella* spp. (8.8%), *Citrobacter* spp. (9.1%) and *Enterobacter* spp. (7.9%).

The carriage of antimicrobial resistance genes was assessed using a DNA microarray previously developed and updated by our group (Batchelor et al., 2008; Anjum et al., 2011; Card et al., 2013). *E. coli* were positive for up to 15 genes detected by the array, while among the non-*E. coli* this comprised up to 12 genes, with the largest number of genes detected in *Citrobacter* spp. Sixty percent of the isolates tested were positive for at least one AMR gene present on the array. Those harboring no detectable resistance genes by array included 305 isolates present on minocycline containing plates, collected from all groups and 109 isolates present on amoxicillin containing, plates collected from all groups.

**Figure 1:**
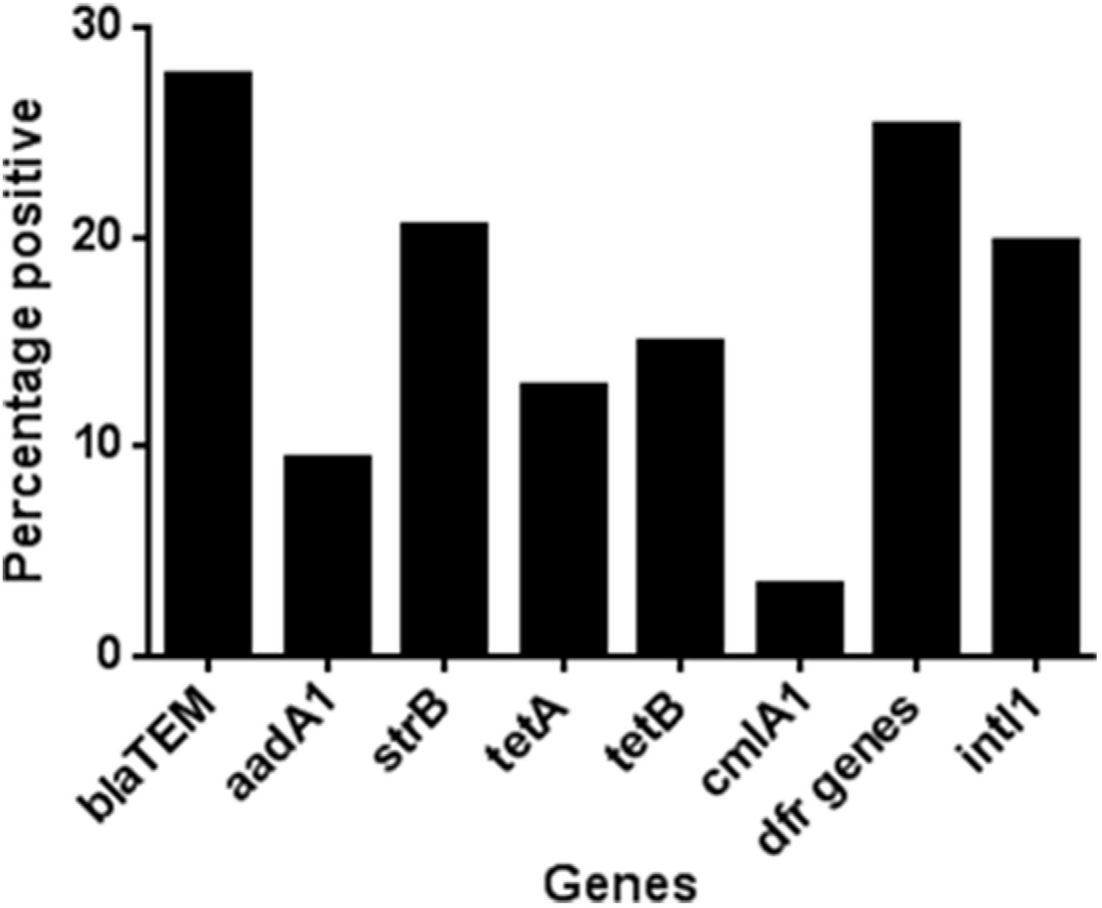
**The percentage of isolates carrying the most common antibiotic resistance associated genes detected during the study**. A total of 1039 isolates were tested, 413 of these isolates were collected from volunteers given a placebo, 378 from those given amoxicillin and 248 from those given minocycline.

The most frequently detected genes in all species are shown in Figure 1. All these genes were more frequently associated with *E. coli*. The exception to this was the gene encoding the AmpC-type enzyme CMY, which was detected in 64% of the *Citrobacter* spp. (*C*. *freundii, C. braakii, C. youngae*, and *C*. *koseri*). Overall i.e., from all time points in the study, 36.9% of all isolates had a MDR genotype (see Methods for definition); while approximately 50% of all the *E. coli* tested had an MDR genotype.

#### Effect of antibiotic administration on resistance gene carriage

**Figure 2:**
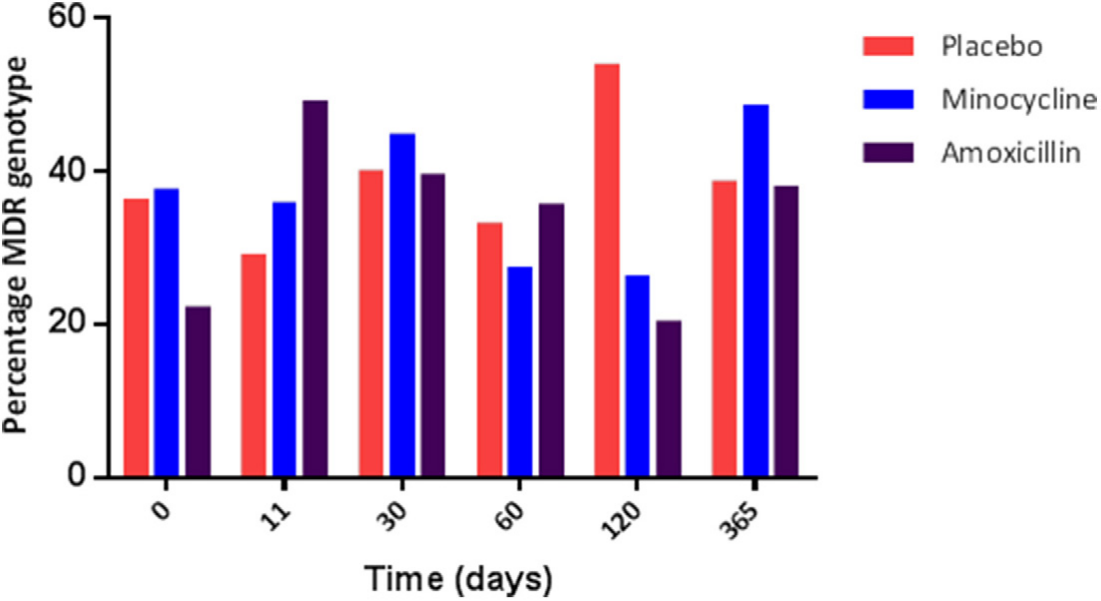
**The proportion of genotypically multi-resistant (i.e., carrying genes for 2 or more resistance gene class) isolate in each treatment group during the study**.

In the minocycline group 36.7% of the total isolates recovered, from all time points in this group, carried genes for two or more antibiotic classes. In the amoxicillin and placebo groups this was, 35.5 and 38.5%, respectively. To assess any effect of antibiotic administration, we determined the change over time in the proportion of isolates with MDR genotype. In the amoxicillin group the proportion of isolates with MDR genotype increased between days 0 and 11 from 22 to 49% (Figure 2), which matched the phenotypic results obtained using a panel of 22 antimicrobial agents or combinations using the VITEK system (data not shown). In the minocycline administration group the proportion of isolates with a MDR genotype did not change between day 0 and 11 (36 and 38%, respectively; Figure 2) and in the placebo group, the proportion of isolates with an MDR genotype was on average 38.5%, for the entire study (range 29–54%); both matched the respective phenotypic data (data not shown). The wide range in the placebo was the effect of an increase in MDR isolates at 120 days to 54% from between 30 and 40% in first four and last sampling point; the cause of this increase was unknown.

**Figure 3:**
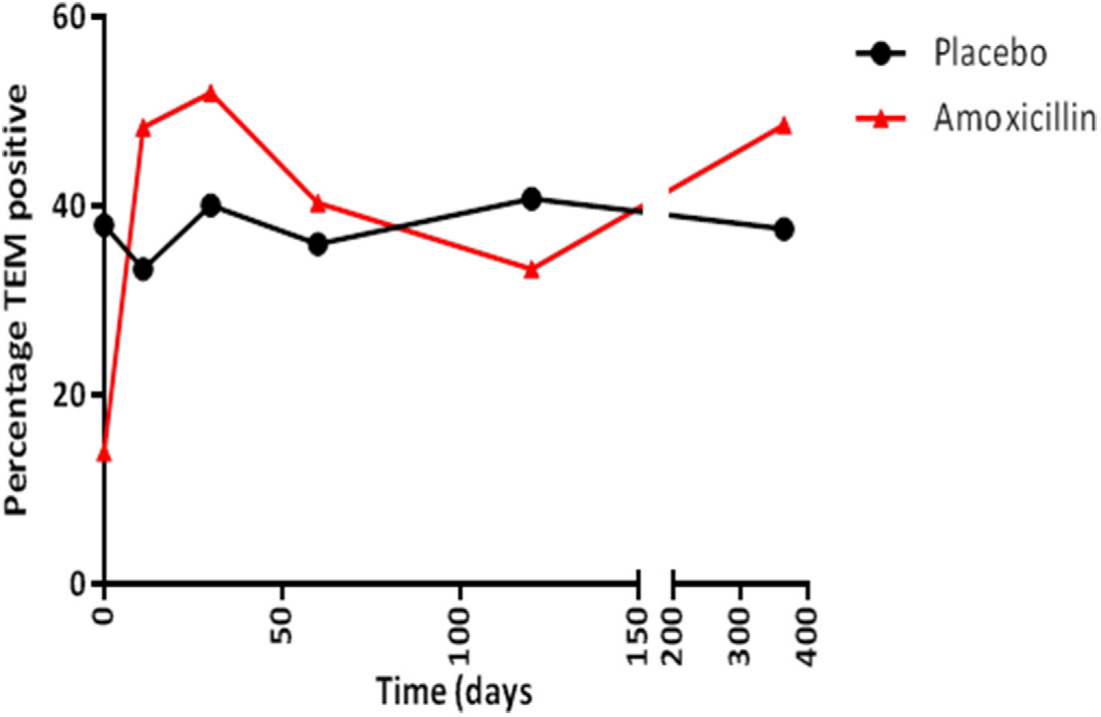
**The percentage of *bla*_TEM_ positive *E. coli* collected from amoxicillin and placebo treatment groups**.

The increase in the resistance genotype observed in the amoxicillin group was due to an overall increase in the percentage of *bla*_TEM_ positive *E. coli* present in this group from day 0 (13.9%) to day 11 (48.3%), which was sustained for up to 30 days following amoxicillin administration (Figure 3); after this period there was a fall in the level of TEM positive isolates although it did not return to pre-administration levels. However, only half the participants challenged with amoxicillin accounted for this increase, indicating person to person variation (data not shown). An increase in the proportion of *tet*(B) positive *E. coli* (from 8.3 to 20%) was also observed immediately following amoxicillin administration. At 30 days post-administration, the levels of *strB, sul1, intI1, dfr*, and *tet*(A) increased above pre-administration levels; this was probably not a direct effect of ampicillin administration (data not shown). In the placebo group the proportion of *bla*_TEM_ positive *E. coli* remained between 33 and 40% for the entire study. In the minocycline administration group the proportion of *bla*_TEM_ positive *E. coli* decreased from 40 to 22% between day 0 and 11. However, the change was probably due to the small number of *bla*_TEM_ positive isolates present in this group and not an effect of treatment (data not shown).

#### Virulence genes present

The DNA array used in the study also encodes genes associated with *E. coli* virulence (Anjum et al., 2007). Virulence associated genes were detected in 57.7% of the isolates tested in this study, including some non-*E. coli* species. The most commonly detected genes were: *senB* (25.1% of all isolates tested); *iss* (34.1%); *prfB* (20.9%); *iroN* (11.8%); *cdtB* (10.9%); and *mchB, mchC*, and *mchF*, which were present in 10.7, 10, and 13.4% of total isolates, respectively. Also of interest was the detection of two Shiga-toxin (*stx1A*) positive *E. coli* from one participant; the presence of this gene was confirmed by PCR (data not shown).

### Characterization of transient and persistent isolates

#### Selection of isolates and PFGE analysis

During our study we noted in all three groups the presence of *E. coli* isolates with similar or identical AMR gene profiles from different time points. To determine if these isolates represented the same clones, we selected (based on numbers of *E. coli* recovered and their resistance profile), two participants from each administration group. A total of 150 *E. coli* were collected from these participants, and all were analyzed by PFGE. Isolates which were collected from different sampling points, but showed indistinguishable PFGE profiles (i.e., clustered together with >85% similarity using UPGMA analysis) were termed “persistent,” and, those isolates with varied PFGE profiles (i.e., from different time points and did not cluster together by UPMGA analysis; they showed <85% similarity) were termed “transient.”

Forty *E. coli* isolates from the amoxicillin administration group from participants A and B were analyzed by PFGE. Nine persistent *E. coli* with indistinguishable profiles were recovered from A from all six sampling points, between day 0 and day 365, while a cluster of 8 isolates with identical PFGE profiles were collected from B from two sampling points, days 30 and 60, following antibiotic administration. Twenty-three i.e., 57.5% isolates from A and B had unique PFGE profiles, they were considered transient. Fifty-eight *E. coli* from the minocycline challenged participants, C and D, were analyzed by PFGE. Four persistent clusters with indistinguishable PFGE profiles were identified (2 from each participant). Each persistent cluster was represented by between 6 and 11 isolates. The clusters from participant C were detected from four sampling points, day 0 up to day 120, and the two clusters from participant D persisted at two different sampling points, from day 0 to 11, and from day 30 to 120, respectively. Twenty-five isolates i.e., 43.1% of isolates tested from C and D had unique PFGE, and were considered transient. Fifty *E. coli* from placebo participants E and F were analyzed by PFGE. A single cluster of 9 isolates was identified in participant F, which persisted over four sampling points between day 0 and day 120. In participant F, three additional clusters (7 isolates in total) were detected across two visits each. There were no persistent isolates identified from participant E. Thirty-four i.e., 68% of the placebo isolates tested were considered transient.

#### Plasmid characterisation and MLST

Fourteen of the 150 *E. coli* representing 7 transient and 7 different persistent clones (as identified by PFGE) were selected for further analysis. At least 2 persistent and 2 transient isolates were selected from each group (Table 1) for plasmid characterization. The aim was to investigate, whether there were any contributions from resident plasmids and antibiotic administration, which impelled isolates to being transient or persistent. Plasmid profiling determined that the isolates carried up to 6 plasmids ranging in size between 4 and 160 kb; up to 5 replicon types; and between 1 and 5 addiction systems (Table 1). Six isolates contained plasmids which were transferable by conjugation and belonged to a range of incompatibility groups. Conjugative plasmids encoding ampicillin resistance were all positive for *bla*_TEM_, and also carried genes encoding resistance to tetracycline, aminoglycoside, sulphonamide, and trimethoprim (Table 1). However, these molecular approaches did not identify any plasmid associated trait specific to the transient or persistent group, or antibiotic administration.

**Table 1:** **Characteristics of the 14 isolates selected for further analysis**.

**Strain ID**	**Administration group**	**Isolation visit**	**Persistent/Transient**	**Plasmid sizes (base pairs)**	Replicon types^**^	**Addiction systems**	**Conjugative plasmids rate (antibiotic used for selection)**	AMR genotype^**^	**MLST type**	**Phenotype array group**
92	Placebo	2	Persistent	1.25e5	IncFIB, U, FII	ccdAB	Non-conjugative	*cmlA1, dfrA12*	ST95 (ST95 Cplx)	1
120	Placebo	6	Persistent	1.28e5, 1.05e5	**IncB/O**, FIB, U, FII	ccdAB	+(Ampicillin) 4.4 × 10^−7^	***sul2, blaTEM***, *strA*, ***strB***	ST95 (ST95 Cplx)	1
121	Placebo	6	Transient	1.6e5	IncFIB, FII	–	Non-conjugative	*aphA, strA, strB, blaTEM, tet*(B)	ST10 (ST10 Cplx)	2
555	Placebo	2	Transient	4.74e4	IncX1	–	Non-conjugative	*aphA, tetB*	ST2035	2
873	Placebo	5	Transient	6.21e3, 3.3e3	Unknown	–	Non-conjugative	*aadA1, strB, blaOXA1, catA1, dfrA1, tet*(B)	ST167 (ST10 Cplx)	2
289	Minocycline	6	Persistent	1.35e5, 7.37e4	IncFIB, FII	pemK, relE, vagCD, hoksok, srnBC	Non-conjugative	*aadA1, ereA, blaOXA2, blaSHV, sul1, tet*(D)	ST10 (ST10 Cplx)	2
729	Minocycline	2	Persistent	1.38e5	IncFIB, FIA, P, FII	pemK, ccdAB, hoksok, srnBC	+ (Tetracycline) 3.16 × 10^−9^	***aadA4***, ***strB***, *blaOXA2*, ***blaTEM***, *catA1, dfrA12, dfrA19, dfrA7*, ***dfrA17***, ***sul1***, ***sul2***, ***tet*(A)***, intI1*	ST69 (ST69 Cplx)	2
1004	Minocycline	4	Persistent	1.02e5, 7.23e3, 3.18e3	**IncB/O**	ccdAB, pndCA	+(Ampicillin) 4.91 × 10^−6^	*strA*, ***strB***, ***blaTEM***, ***dfrA14***,*drfA15*, ***sul2***	ST10 (ST10 Cplx)	1
502	Minocycline	7	Transient	1.02e5, 8.06e3	IncFIB, FII	ccdAB, srnBC	Non-conjugative	*strA, strB, blaTEM, tet*(B)	ST2076	2
737	Minocycline	3	Transient	1.16e5, 5.26e3	IncFII	ccdAB, srnBC	Non-conjugative	*ereB, blaTEM, catA1, dfrA12, dfrA7/17, sul1, tet*(B)	ST10 (ST10 Cplx)	2
570	Amoxicillin	2	Persistent	1.23e5, 9.99e4	IncN, FIB, FIIS, U,**FII**	ccdAB, hoksok	+(Ampicillin) <2.12 × 10^−7^	*aadA2, blaPER, blaOXA2*, ***blaTEM***, *cmlA1*, ***dfrA12***, *dfrA13, sul1, intI1*	ST404 (ST14 Cplx)	1
1053	Amoxicillin	4	Persistent	1.67e5, 1.22e5, 9.56e4, 4.72e4	**IncI1**, **L/M**, FIB, **R**, FII	pemK, vagCD, pndCA	+(Ampicillin) 1.33 × 10^−7^	*aadA1*, ***aadA2***, *strA*, ***strB***, ***blaTEM***, *sul2, tet*(A), ***tet*(B)**, *intI1*	ST227 (ST10 Cplx)	2
907	Amoxicillin	5	Transient	1.16e4	IncU, **FII**	pemK, hoksok	+(Ampicillin) 6.67 × 10^−9^	***aadA1***, *aadA2, strA*, ***strB***, ***blaTEM***, *dfrA1, sul1, sul2, tet*(A), *intI1*	ST2064	2
1048	Amoxicillin	4	Transient		Unknown	–	Non-conjugative	*aadA1, blaTEM, dfrA1, tet*(A)	ST196	2

** *Replicon types and AMR genes transferred to the transconjugants following conjugation are highlighted in bold. Cplx, clonal complex*.

The MLST types were determined for the 14 selected isolates. Six of the 14 isolates belonged to ST complex 10; the remaining isolates belonged to a variety of ST (Table 1). A specific ST complex was not associated with transient or persistent group, or antibiotic administration.

#### Phenotypic microarrays

**Figure 4:**
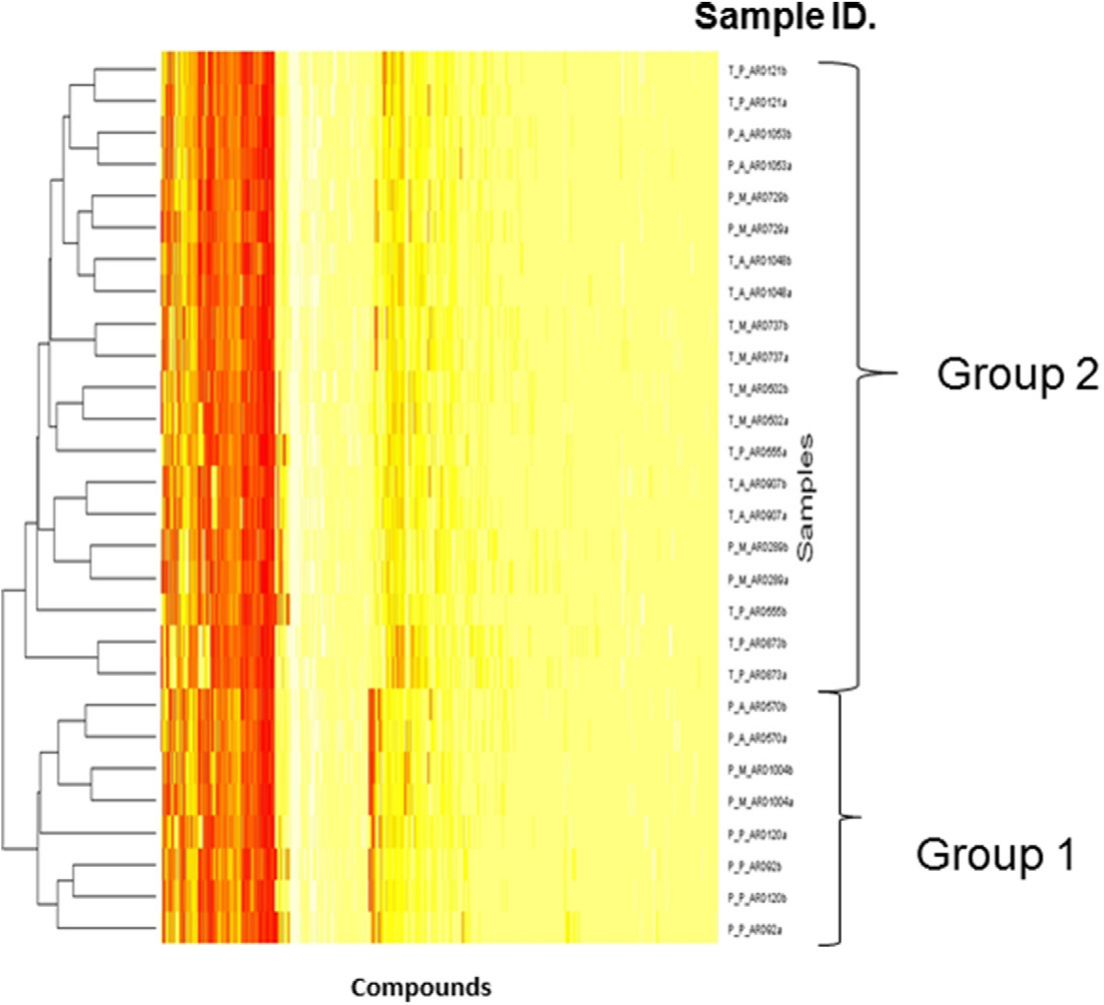
**Dendrogram showing the Biolog results for 14 selected isolates representing transient and persistent isolates**. Each experiment was repeated in duplicate and both replicates are represented individually in the figure. The two groups generated by comparing the respiration profiles of the isolates are indicated. The Hierarchical clustering was used to generate a dendrogram using R-conductor program (http://www.bioconductor.org/). The sample ID is defined as such: first T or P indicating if a strain is transient or persistent, the second letter (A, M, P) indicates which treatment was administered and the final ARD reference is the isolate number.

Biolog phenotype microarrays were utilized to determine whether there were conditions which could help explain why some isolates were transient and others persistent, and if there was an effect of antibiotic administration. The 14 isolates selected above were exposed to different stress modulators and their ability to respire in the conditions measured (Supplementary Table 2). A hierarchical clustering was performed on the results of respiration (AUC values) of isolates using compounds present on all 10 plates (Figure 4). In general, technical replicates showed high similarity, but it was not possible to distinguish between isolates from the minocycline, amoxicillin, and placebo groups using this phenotypic method. However two groups of isolates were detected by hierarchical clustering; group 1 contained 4 isolates and group 2 contained 10 isolates. All seven transient isolates grouped within group 2, while persistent isolates were divided between groups 1 and 2 (Figure 4). A principal component analysis (PCA) was also performed on the results of respiration (AUC values) and identified the same two groups shown in Figure 4 (data not shown). Isolates in each group did not represent any single ST type or complex. Several compounds were identified in which all or most isolates within group 2 were able to respire (Table 2). These included several antiseptic biocidal agents such as domiphen bromide and alexidine, and two compounds which are known to modulate efflux mechanisms, chlorpromazine and promethazine (Table 2). Surprisingly, all isolates from group 2 showed the presence of tetracycline resistance genes by array (Table 1), which were absent from all group 1 isolates; as a result differential respiration rates were observed between group 1 and group 2 for several tetracycline derivatives (Table 2).

**Table 2:** **Compounds identified using the Biolog for which differential respiration levels were detected between groups 1 and 2**.

**Compound**	**AUC values**		**Adjusted *P*-value**
	**Group 1**	**Group 2**	
Tetracycline derivatives (e.g., Doxycycline)	11.74	14.61	0
Chlorpromazine	11.82	13.42	0
Promethazine	11.90	13.65	0.003
Alexidine	12.40	14.15	0.015
Domiphen bromide	11.40	13.30	0.023

## Discussion

Here we describe the results of a yearlong study in which healthy individuals were given a course of antibiotics, and an analysis performed on the Gram-negative fecal facultative anaerobic bacteria collected during the study. Although 18 genera of bacteria were detected, the predominant bacterial species detected in the facultative anaerobic gut flora was *E. coli*; *Klebsiella, Citrobacter*, and *Enterobacter* were also prevalent. It is known that the facultative anaerobic flora is the subdominant population in the gut with the dominant population consisting mainly of members of the *Firmicutes* and *Bacteroidetes* phyla (Eckburg et al., 2005). Nevertheless, the gastrointestinal tract can contain between 10^2^ and 10^10^ Enterobacteria, with the highest proportion being in the feces (Simon and Gorbach, 1984).

Identification of trends in the results was difficult as the base-line level of resistance genes, i.e. resistance at time point zero, was high in all groups including the placebo. On average, 58% of all isolates tested carried at least one resistance gene (detectable by array) prior to treatment (62% minocycline, 50.7% amoxicillin, 61% placebo) and in the placebo group some isolates carried up to 11 resistance genes at the start of the study. This demonstrated that generally the levels of resistance gene carriage in the gut flora were high in the healthy adults randomly selected for this study; we were unable to detect the cause for this. However, it is noteworthy that for some participants we did not detect any resistance genes throughout the study, while for other participants the number of resistance genes fluctuated during the study; although this was irrespective of antibiotic administration it was particularly apparent in the placebo group (data not shown).

Nevertheless, it was clear from our results that following the administration of amoxicillin the levels of *bla*_TEM_ gene positive *E. coli* isolates (*bla*_TEM_ gene confers resistance to amoxicillin) increased, suggesting this antibiotic was selecting for these isolates; the levels remained above the pre-challenge levels throughout the remainder of the study. A small increase in tetracycline resistance genes was also noted post-amoxicillin administration, linked to the increase in resistant *E. coli*, indicating co-selection. A similar increase was not seen in the other treatment or the placebo group for any facultative anaerobic bacteria. Also, amoxicillin use did not have the same effect on any of the other beta-lactamase resistance genes detectable by the array. The increase in *bla*_TEM_ positive *E. coli* isolates following amoxicillin use could be due to the transfer of *bla*_TEM_ to sensitive *E. coli* and/or the proliferation of *bla*_TEM_ *E. coli* isolates as they are better able to survive. The high percentage similarity in *E. coli* from the PFGE data, from two participants in the amoxicillin group, supports proliferation of *bla*_TEM_ positive *E. coli* (data not shown). However, the conjugation experiments demonstrated that *bla*_TEM_ was contained on transferable plasmids, which harbor other resistances including tetracycline (Table 1). Therefore, the increase seen post amoxicillin use could reflect spread of a multi-resistant plasmid in some participants and proliferation of a multi-resistant *E. coli* in others. The potential of plasmids to spread between bacteria in the gut is already known and has been demonstrated using *Salmonella* and *E. coli* in a gut mouse model (Stecher et al., 2012).

Extended spectrum beta-lactamase resistance was rare in this study, with only 3.7 and 2.9% of all isolates tested demonstrating resistance to cefotaxime or ceftazidime, respectively. The *bla*_CTX−M_ genes were present in only 20 of the *E. coli* tested, which were from two participants and carried either *bla*_CTX−M−14_ or *bla*_CTX−M−15_. Sommer et al. (2009) have also demonstrated the presence of *bla*_CTX−M_ during analysis of the resistome in healthy volunteers (Sommer et al., 2009). It is known that *bla*_TEM_ can be prevalent among *E. coli* and can have a wide-spectrum of beta-lactamase activity when mutations are introduced (Bush and Jacoby, 2010), but within this study we did not determine the spectrum of activity for the TEM enzymes.

Analysis of the virulence genes carried by the *E. coli* isolates demonstrated that although these *E. coli* are thought to be “commensals” isolated from healthy individuals, several harbored genes with distinct virulence characteristics associated with different *E. coli* pathotypes. For example, the presence of the *iss* gene has been associated with extra-intestinal pathogenic *E. coli* and encodes a protein involved in serum survival (Johnson et al., 2008); while *senB* is a plasmid encoded enterotoxin gene associated with Entero-invasive *E. coli* (Nataro et al., 1995). Genes associated with Entero-pathogenic, Entero-toxigenic, and Entero-aggregative *E. coli* were also detected during this study (Anjum et al., 2007). In addition, two Shiga-toxin positive *E. coli* (STEC) isolates were detected from an apparently healthy participant. Both STEC strains carried *stx1* and lacked the *eae* gene which encodes an adhesion protein involved in enterocyte attachment; characteristics that have been associated with less virulent STEC (Beutin et al., 2004), which are less likely to elicit the severe symptoms seen in infections associated with highly pathogenic *E. coli* O157 (Friedrich et al., 2002; Anjum et al., 2007, 2014).

Comparison of the *E. coli* PFGE profiles demonstrated that isolates with identical PFGE profiles were detected at several sampling points demonstrating the persistence of the same clone; in some instances these clones were detected for the entire study. In this study bacteria were selected based on colony morphology following their initial selection on antibiotic plates, therefore the isolates selected are likely to represent a snapshot of the most prevalent population/clone at the time of the sampling. Conversely, those isolates that showed unique PFGE profiles (i.e., transient) and were detected at one sampling point, were unable to persist, or at least were not the most prevalent clone at other sampling points and hence remained undetected. A larger sample size or more frequent sampling may have increased the probability of detecting transient isolates that maybe present. In many instances multiple colonies from the same sampling point showed the same AMR and PFGE profiles, indicating the same clones had been selected on multiple occasions.

Following the identification of persistent and transient *E. coli* isolates, it was of interest to know whether there were any plasmid characteristics that distinguished these groups, enabling establishment of the former in the human gut, over the latter. Plasmid analysis, using methods employed in this study, demonstrated that there were no unique characteristics that could be used to distinguish between plasmids from isolates considered persistent and transient. However, the most common MLST complex detected, including both persistent and transient isolates was ST complex 10, which has been reported throughout the world in humans and animals (http://mlst.ucc.ie/mlst/dbs/Ecoli/).

The PM Biolog system was used to assess differences in bacterial fitness/survival in the presence of different stress modulators, including sub lethal doses of antibiotics, in selected persistent and transient isolates from the 3 different treatment groups. Although it was not possible to distinguish between isolates from the 3 groups with this method, we were able to distinguish between some isolates that persisted in the gut and those that appeared transient. All transient isolates selected were better able to metabolize several antiseptic biocidal compounds, which could help in their ability to survive in different conditions. These isolates were also all resistant to tetracycline. Ciric et al. (2011) have reported the presence of a Tn*916*-like element from *Streptococcus oralis* isolated from a human oral cavity to harbor both a tetracycline resistance gene and an antiseptic resistance protein. Therefore, the tetracycline and biocidal resistant genes may be present on a mobile element in all transient and some persistent isolates. But several persistent *E. coli* isolates had possibly lost this mobile element as they adapted to a stable gut environment, and thereby, lost the ability to metabolize the biocidal compounds.

It is worth noting that 20% of the 1039 isolates examined were not fully resistant to the antibiotics used for their selection, the majority being from minocycline selection plates. This is probably because these isolates were not clinically resistant. There is no clinical breakpoint set by EUCAST for minocycline in *Enterobacteriaceae*, although historically BSAC have suggested 0.5 mg/L and CSLI 4 or 8 mg/L (http://www.eucast.org/fileadmin/src/media/PDFs/EUCAST_files/Rationale_documents/Minocycline_Rationale_Document_1.0_20091202.pdf). The epidemiological cut-off (ECOFF) for minocycline differs depending on species and is in the range of 0.25 mg/L to 64 mg/L. For *E. coli* the ECOFF is 4 mg/L, and 8 mg/L for *Klebsiella* spp. and *Citrobacter* spp., and the selection plates contained either 0.5 mg/L minocycline or 4 mg/L minocycline. Also, many non*-E. coli* isolates, which were recovered on the amoxicillin plates, harbored no detectable resistance genes by array. It is likely that for some of the non-*E. coli* recovered the genes responsible for resistance in these species were not represented on the array, or they may have been intrinsically resistant to the selective agent, or they were protected by growing adjacent to a resistant organism.

In conclusion, this study has highlighted that there is a diverse range of resistance genes circulating in the gut of healthy individuals in the absence of selective pressures, including those such as *bla*_CTX−M_, which are of clinical concern. We showed that the use of antibiotics can have a temporary effect on the resistance genes present. In addition, the detection of virulence genes in *E. coli* indicates that not all *E. coli* present in the human gut are innocuous commensals, and it is common for *E. coli* to persist in the gut for different time periods, which may result in loss of genes and adaptation to the gut environment.

### Conflict of interest statement

The authors declare that the research was conducted in the absence of any commercial or financial relationships that could be construed as a potential conflict of interest.
