# Supplementary material for: Antimicrobial resistance characteristics and fitness of Gram-negative fecal bacteria from volunteers treated with minocycline or amoxicillin
Source: Front Microbiol. 2014 Dec 17;5:722. doi: 10.3389/fmicb.2014.00722 (PMC4269195; doi:10.3389/fmicb.2014.00722)
Supplement: Supplementary file 1 [file Table1.DOCX]

**Supplementary Table 1.**

Table showing the different bacterial species identified during this study and the number of isolates of each species. (Note; the table only includes those isolates for which data has been included in the paper. Data was excluded from participants who dropped out of the study and data from *Bacillus*, *Candida*, *Enterococcus* isolates was also excluded).

| **Bacterial Genera** | **Number of isolates** | **Species (number of isolates for each species)** |
| --- | --- | --- |
| *Escherichia* | 739 | *E. coli* |
| *Acinetobacter* | 1 | *A. guillouiae* |
| *Klebsiella* | 95 | *K. pneumoniae* (60) *K. oxytoca* (31) |
| *Kluyvera* | 2 | *K. ascorbata* |
| *Citrobacter* | 103 | *C. amalonaticus* (1), *C. braakii* (9), *C. youngae* (1), *C. farmeri* (2), *C. freundii* (71), *C. gillenii* (3), *C. koseri* (7). *C. murliniae* (1) |
| *Comamonas* | 1 | *C. kerstersii* |
| *Cronobacter* | 1 | *C. sakazakii* |
| *Delftia* | 2 | *D. acidovorans* |
| *Enterobacter* | 82 | *E. aerogenes* (7), *E. amnigenus* (1), *E. asburiae* (3), *E. cloacae* (64), *E. hormaechei* (2), *E. kobei* (3), *E. ludwigii* (2) |
| *Hafnia* | 7 | *H. alvei* |
| *Morganella* | 2 | *M. morganii* |
| *Pantoea* | 1 |  |
| *Proteus* | 1 | *P. mirabilis* |
| *Pseudomonas* | 11 | *P. aeruginosa* (7) *P. putida* (2), *P. spp*. (1) |
| *Raoultella* | 8 | *R. ornithinolytica* (6), *R. terrigena* (2) |
| *Rhizobium* | 1 | *R. radiobacter* |
| *Stenotrophomonas* | 3 | *S. maltophilia* (2), *S. acidaminiphila* (1) |
| *Sphingomonas* | 1 | *S. paucimobili* |
| *Yersinia* | 2 | *Y. enterocolitica* |
